# Valproic Acid Induces Hair Regeneration in Murine Model and Activates Alkaline Phosphatase Activity in Human Dermal Papilla Cells

**DOI:** 10.1371/journal.pone.0034152

**Published:** 2012-04-10

**Authors:** Soung-Hoon Lee, Juyong Yoon, Seung Ho Shin, Muhamad Zahoor, Hyoung Jun Kim, Phil June Park, Won-Seok Park, Do Sik Min, Hyun-Yi Kim, Kang-Yell Choi

**Affiliations:** 1 Translational Research Center for Protein Function Control, Department of Biotechnology, College of Life Science and Biotechnology, Yonsei University, Seoul, South Korea; 2 Skin Research Team, Skin Research Institute, Amore Pacific Corporation Research and Development Center, Kyounggi-do, South Korea; 3 Department of Molecular Biology, College of Natural Science, Pusan National University, Busan, South Korea; Brunel University, United Kingdom

## Abstract

**Background:**

Alopecia is the common hair loss problem that can affect many people. However, current therapies for treatment of alopecia are limited by low efficacy and potentially undesirable side effects. We have identified a new function for valproic acid (VPA), a GSK3β inhibitor that activates the Wnt/β-catenin pathway, to promote hair re-growth *in vitro* and *in vivo*.

**Methodology/ Principal Findings:**

Topical application of VPA to male C3H mice critically stimulated hair re-growth and induced terminally differentiated epidermal markers such as filaggrin and loricrin, and the dermal papilla marker alkaline phosphatase (ALP). VPA induced ALP in human dermal papilla cells by up-regulating the Wnt/β-catenin pathway, whereas minoxidil (MNX), a drug commonly used to treat alopecia, did not significantly affect the Wnt/β-catenin pathway. VPA analogs and other GSK3β inhibitors that activate the Wnt/β-catenin pathway such as 4-phenyl butyric acid, LiCl, and BeCl_2_ also exhibited hair growth-promoting activities *in vivo*. Importantly, VPA, but not MNX, successfully stimulate hair growth in the wounds of C3H mice.

**Conclusions/ Significance:**

Our findings indicate that small molecules that activate the Wnt/β-catenin pathway, such as VPA, can potentially be developed as drugs to stimulate hair re-growth.

## Introduction

Alopecia causes serious problems for numerous people in the world. However, current therapies for the treatment of alopecia are limited by low efficacy and potentially undesirable side effects caused by application of available drugs [1]. The Wnt/β-catenin pathway is important for several developmental processes, including hair follicle development [2]–[4]. Activation of the Wnt/β-catenin pathway is required for initiation of hair follicle formation [5], and stimulates growth and differentiation of hair by maintaining expression of genes that function at the anagen phase of the hair cycle [6]. Wnt3a and Wnt7a maintain dermal papillae in anagen phase as inductive signals for hair growth [6]. In addition, β-catenin accumulates in the nuclei of dermal papillae during anagen, and provides growth signals for hair follicle progenitors [7], [8]. Finally, the Wnt/β-catenin pathway plays a role in the differentiation of stem cells into hair follicular keratinocytes [9]. Together, these findings indicate that the Wnt/β-catenin pathway could be an ideal target for the development of drugs that activate hair growth by inducing anagen phase genes. Unfortunately, there are currently no known drugs that stimulate hair growth by targeting the Wnt/β-catenin pathway.

Valproic acid (VPA; 2-propyl-pentanoic acid) is a mood stabilizer commonly prescribed for the treatment of epilepsy and bipolar disorders over the last several decades [10], [11]. VPA is known as a histone deacetylase inhibitor that exerts its effects through modification of chromatin structure and gene expression [12], [13]. VPA is also known to affect several different signaling pathways including protein kinase C, extracellular signal-regulated kinase (ERK), and Wnt/β-catenin pathways [14]–[16]. VPA activates the Wnt/β-catenin pathway by inhibiting GSK3β [17], [18], leading to axonal remodeling, synaptic protein clustering, and differentiation of neuronal progenitors [19], [20].

We investigated the effect of VPA on the hair re-growth of C3H mice by considering the relationship between the Wnt/β-catenin pathway and hair formation. VPA induced hair re-growth as efficiently as MNX and activated the Wnt/β-catenin pathway *in vivo*. The potential importance of VPA in human hair re-growth was indicated by the increased expression of ALP and activation of the Wnt/β-catenin pathway following treatment of VPA in human dermal papilla cells. Alkaline phosphatase (ALP), a prominent dermal papilla marker [21]–[23], was identified as a critical marker for hair growth promotion. The role of the Wnt/β-catenin pathway in the regulation of ALP activity was further investigated by testing VPA analogs and other Wnt activators *in vivo* and *in vitro*. Finally, we further characterized specific roles of VPA in induced hair growth in the wounds of C3H mice. These VPA effects accompanying the activation of keratin 15 and CD34 stem cell markers also correlate with the Wnt/β-catenin pathway and ALP activities.

## Materials and Methods

### Reagents

VPA, 4-phenyl butyric acid (PBA), and 2-ethyl butyric acid (EBA) were purchased from Acros. Lithium chloride (LiCl) and beryllium chloride (BeCl_2_) were purchased from Sigma, and MNX was purchased from Daejung.

### Animals and *in Vivo* Hair Growth Test

Six-wk-old male C3H mice were purchased from Orient Bio Co., and allowed to adapt to their new environment for 1 wk. The hairs on the backs of 7-wk-old mice, whose hair follicles were in telogen phase, were shaved with a hair clipper and 300 µl of each drug at an appropriate concentration (as described in figure legends) was applied topically daily for up to 28 d. All reagents used for the hair re-growth test were dissolved in a vehicle composed of 50% (vol/vol) ethanol, 30% water, and 20% propylene glycol. TOP-GAL transgenic mice carrying the *lac Z* reporter gene downstream of the *c-fos* promoter that responds to the lymphoid enhancer binding factor 1/transcription factor 3 (LEF/TCF) mediated signaling pathway and activated β-catenin were purchased from the Jackson Laboratory. Genotyping was confirmed by standard polymerase chain reaction (PCR) using the following primers: transgene forward 5′-ATCCTCTGCATGGTCAGGTC-3′, transgene reverse 5′-CGTGGCCTGATTCATTCC-3′; internal positive control forward 5′-CAAATGTTGCTTGTCTGGTG-3′, internal positive control reverse 5′-GTCAGTCGAGTGCACAGTTT-3′. All animal procedures were approved by the Institutional Review Board of Severance Hospital, Yonsei University College of Medicine (09-013).

### Quantitative Histomorphometry

Hair cycle stages were evaluated and classified as described [24] by the means of quantitative histomorphometry. Histomorphomety was performed with hematoxylin and eosin (H&E)-stained sections that were taken from defined back skin regions. At least 50 hair follicles per mouse per group were evaluated.

### Cell Culture

Human dermal papilla cells were obtained from Dr. Jin-Ho Jung in the Department of Dermatology at Seoul National University, and maintained in Dulbecco′s Modified Eagle Medium (DMEM) containing 10% (vol/vol) fetal bovine serum (FBS) and supplemented with 0.1 mg/ml G418 (Gibco-BRL). Cells were plated at a density of 6×10^5^ cells per 10-cm diameter dish.

### Western Blotting Analysis

Cells or tissue that was ground in mortars were lysed in RIPA buffer (150 mM NaCl, 10 mM Tris, pH 7.2, 0.1% sodium dodecyl sulfate (SDS), 1.0% Triton X-100, 1% sodium deoxycholate, 5 mM EDTA). Samples were separated on 10–12% SDS polyacrylamide gels and transferred onto a PROTRAN®nitrocellulose membrane (Schleicher and Schuell Co.). After blocking in phosphate buffered saline (PBS) containing 5% non-fat dry skim milk and 0.07% (vol/vol) Tween 20, the blots were incubated with the following primary antibodies overnight at 4°C: anti-β-catenin (Santa Cruz Biotechnology, 1∶1000), p-Erk (Santa Cruz Biotechnology, 1∶1000), p-Akt (Santa Cruz Biotechnology, 1∶1000), fillagrin (Covance, Berkeley, CA, 1∶1000), loricrin (Covance, 1∶1000), keratin 14 (Covance, 1∶1000), α-tubulin (Oncogene Research Products, 1∶5000), PCNA (proliferating-cell nuclear antigen, Santa Cruz Biotechnology, 1∶500), BMP4 (R&D systems, 1∶500), and ALP (Abcam, 1∶100). Horseradish peroxidase-conjugated anti-mouse (Cell Signaling) and anti-rabbit (Bio-Rad) antibodies were used as secondary antibodies. The signals were detected by enhanced chemiluminescence (Amersham Bioscience) using a luminescent image analyzer, LAS-3000 (Fujifilm).

### Reverse Transcription (RT)-PCR

Total RNA was prepared using TRIzol reagent (Invitrogen) and cDNA was synthesized *in vitro* using M-MLV reverse transcriptase (Invitrogen). PCR was performed with Taq DNA polymerase using a System 2700 (Applied Biosystems) at 94°C for 5 min, followed by 25–30 cycles of 94°C for 30 sec, 55–60°C for 1 min, and 72°C for 1 min. The PCR products were electrophoresed on agarose gels and photographed using a LAS-3000 analyzer. The following primer sets were used: filaggrin, forward 5′-GCTTAAATGCATCTCCAG-3′ and reverse 5′-AGTCAGTCCTATTGCAGG-3′; loricrin, forward 5′-CCTACCTGGCCGTGCAAG-3′ and reverse 5′-CATGAGAAAGTTAAGCCCATCG-3′; keratin 14, forward 5′-GGACGCCCACCTTTCATCTTC -3′ and reverse 5′- ATCTGGCGGTTGGTG GAGG-3′; GAPDH, forward 5′-ACCACAGTCCATGCCATCAC-3′ and reverse 5′-TCCACCACCCTGTTGCTGTA-3′; BMP4, forward 5′-AGCAGCCAAACTATGGGCTA-3′ and reverse 5′-TGGTTGAGTTGAGGTGGTCA-3′; BMP6, forward 5′-AACCAACCACGCGATTGTG-5′ and reverse 5′-AAGTCTCATCGTCCCACCTC-3′; and ALP, forward 5′-CAAACCGAGATACAAGCACTCCC-3′ and reverse 5′-CGAAGAGACCCAATAGGTAGTCCAC-3′.

**Figure 1:**
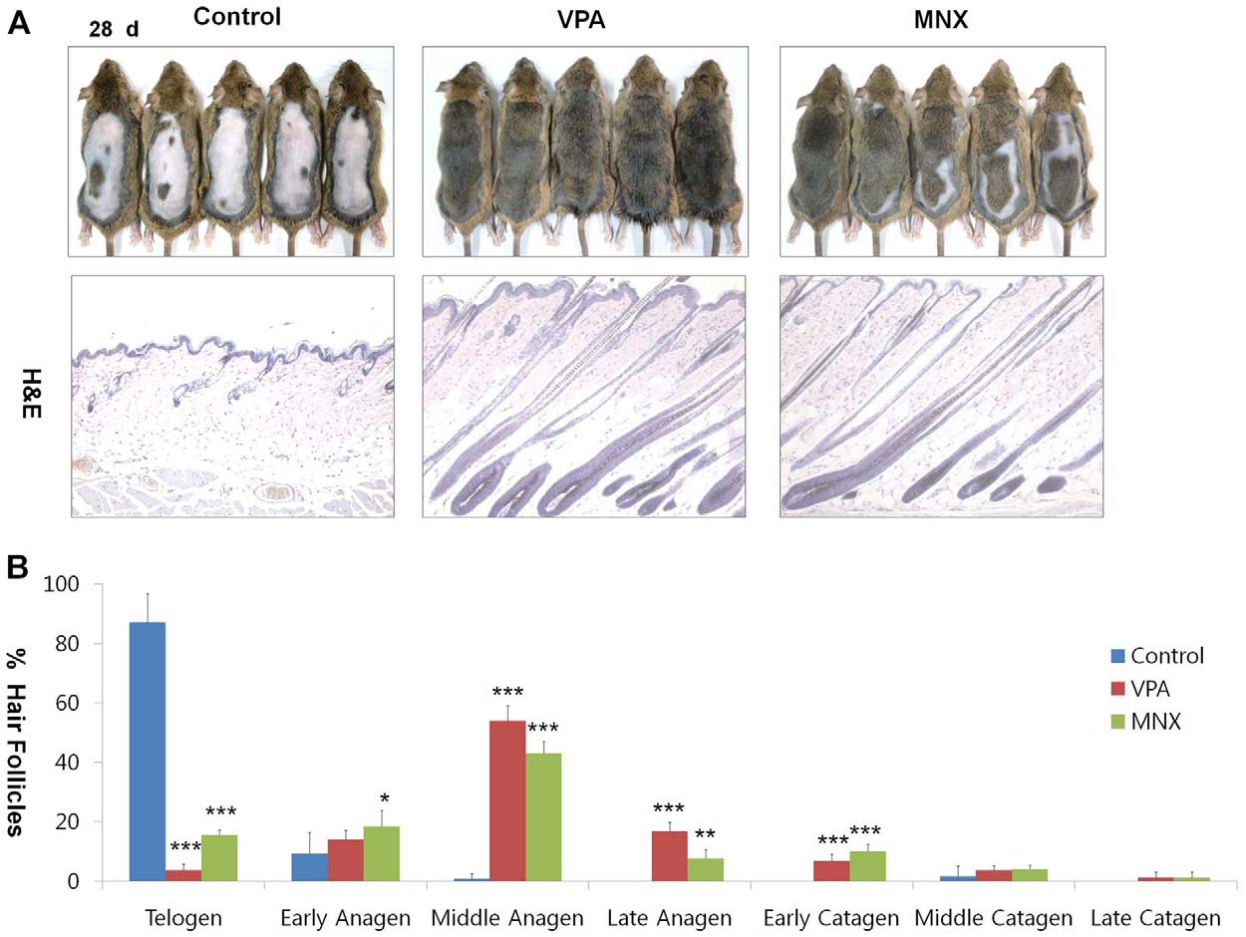
Effects of VPA and MNX on hair re-growth, hair cycle stage in mouse skin. The back skin of 7-wk-old male C3H mice was shaved and treated daily with topical application of vehicle, 500 mM valproic acid (VPA), or 100 mM minoxidil (MNX) for 28 d. (A) Gross images showing hair re-growth in C3H mice treated with VPA or MNX for 28 d (upper panel) and H&E staining of the VPA or MNX-treated skin for 28 d (lower panel). (B) Quantitative histomorphometic analyses of skin tissues treated with VPA or MNX for 28 d were performed with 5 mice for each group; Early anagen, anagen I-anagen II; Middle anagen, anagen III-IV; Late anagen, anagen V-anagen VI; Early catagen, catagen I-catagen III; Middle catagen, catagen IV-V; Late catagen, catagen VI-catagen VIII. Asterisks denote significant differences between control and test groups as measured by t-test with one asterisk being p<0.05, two asterisks being p<0.005, and three asterisks being p<0.0001. Original magnification: A, ×100 (H&E staining).

**Figure 2:**
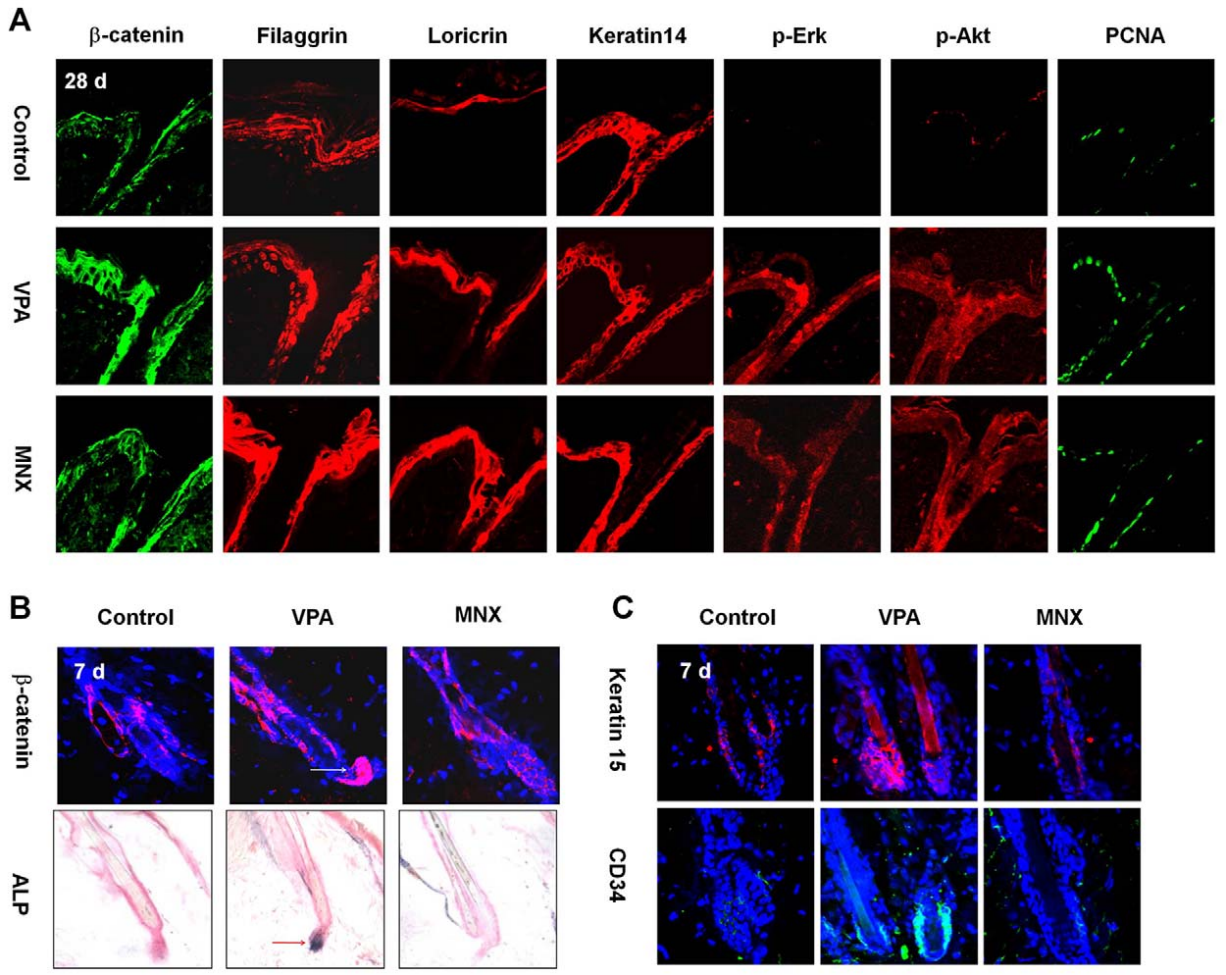
Effects of VPA and MNX on epidermal differentiation markers, ALP activities, and hair follicular stem cell markers. The back skin of 7-wk-old male C3H mice was shaved and treated daily with topical application of vehicle, 500 mM valproic acid (VPA), or 100 mM minoxidil (MNX) for 7 d or 28 d. (A) Immunohistochemical analysis of epidermis of C3H mice treated with VPA or MNX for 28 d with antibody against β-catenin, filaggrin, loricrin, keratin 14, p-Erk, p-Akt, or PCNA. (B) Immunohistochemical analysis of β-catenin expression in hair follicles (upper panel) and ALP staining (lower panel) of skin treated with vehicle, VPA, or MNX for 7 d. Dark blue region in the dermal papillae of hair follicles (a red arrow in middle panel) represent positivity for ALP activity. (C) Immunohistochemical analysis was performed with anti-keratin 15, or -CD34 antibody. Original magnification: A, ×635; B, ×635 (immunohistochemistry) and ×400 (ALP staining); C, ×635.

**Figure 3:**
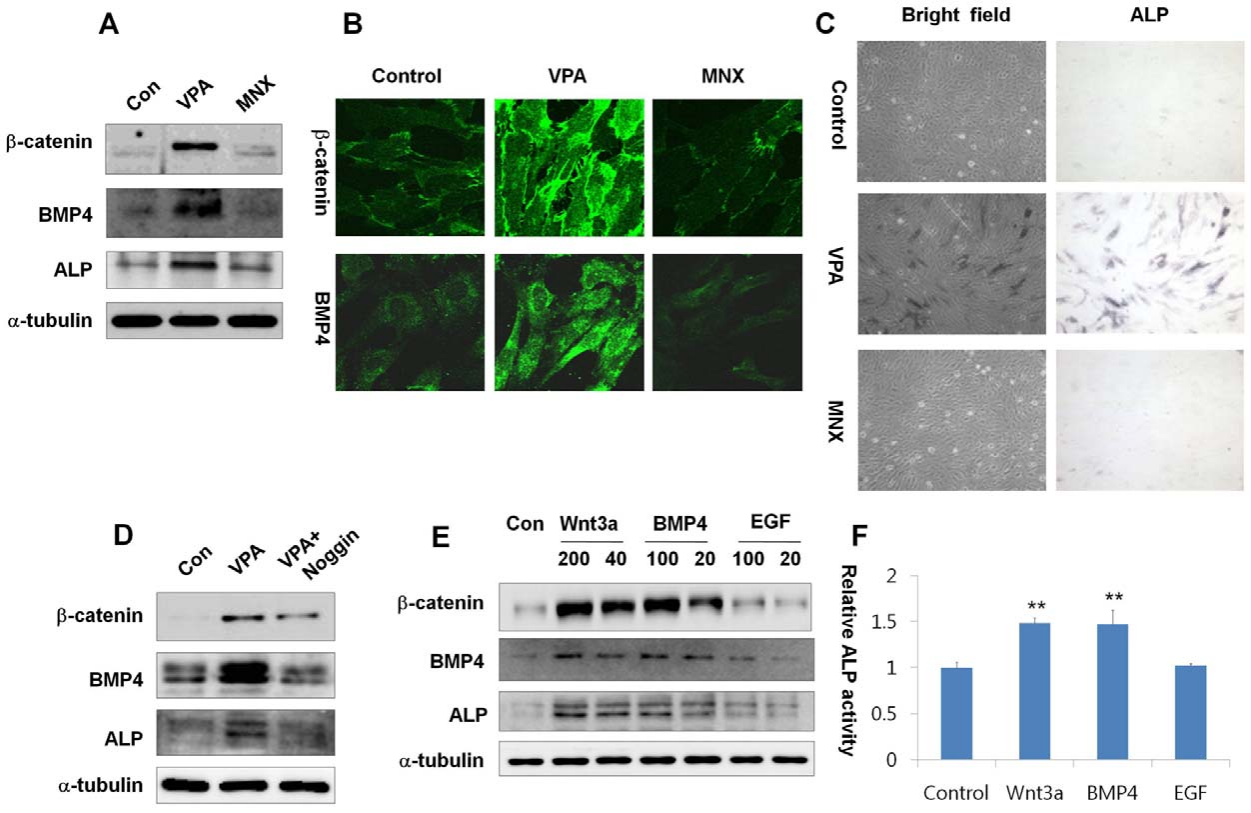
Effects of VPA and MNX on the activation status of the Wnt/β-catenin pathway and ALP activity in human dermal papilla cells. Human dermal papilla cells at passage 11 with minimal ALP activity were used to test the ability of VPA and MNX to recover ALP activity. Cells were grown in DMEM supplemented with 10% heat-inactivated FBS, G418 (100 µg/ml), streptomycin (100 µg/ml), and penicillin G sodium (100 µg/ml) in 5% CO_2_ at 37°C, and treated with 1 mM VPA or 100 µM MNX for 72 h. (A) Western blotting for β-catenin, BMP4, ALP and α-tubulin. (B) Immunocytochemical staining with antibody against β-catenin or BMP4. (C) ALP staining. Cell morphology (left panels) was examined under a bright-field microscope. Dark blue staining indicates ALP-expressing cells (right panels). (D) Western blotting for β-catenin, BMP4, ALP, and α-tubulin in human dermal papilla cells treated with VPA or noggin (500 ng/ml). (E) Western blotting for β-catenin, BMP4, ALP, and α-tubulin expression in human dermal papilla cells treated with Wnt3a (200 or 40 ng/ml), BMP4 (100 or 20 ng/ml), or EGF (100 or 20 ng/ml) for 72 h. (F) ALP activity was measured as described in Methods after treatment with Wnt3a (200 ng/ml), BMP4 (100 ng/ml), or EGF (100 ng/ml) for 72 h. Asterisks denote significant differences between control and test group as measured by t-test with one asterisk being p<0.05 and two asterisks being p<0.005. Original magnification: B, ×635; C, ×100.

**Figure 4:**
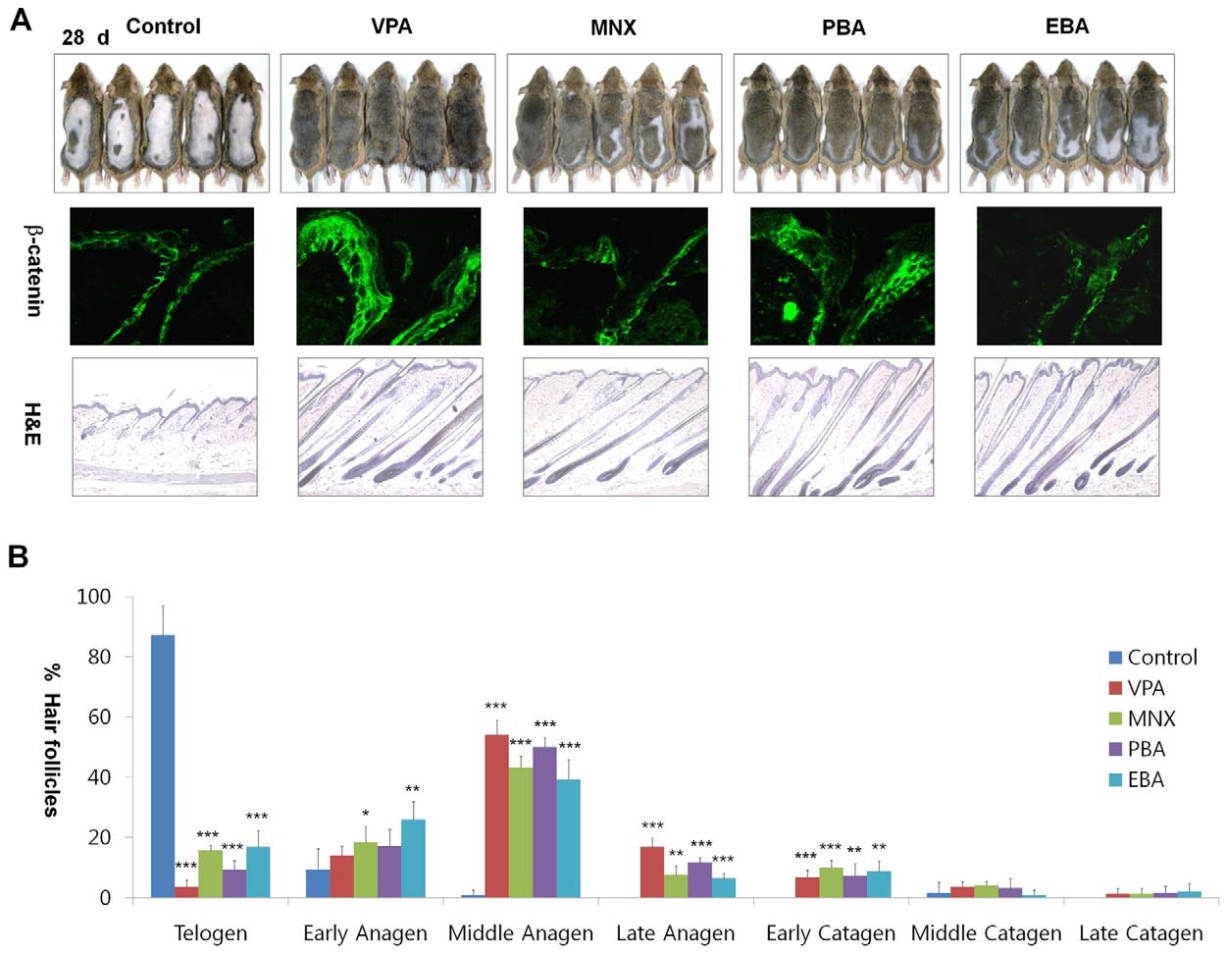
Effects of Wnt/β-catenin pathway activators on hair re-growth on mouse skin. The vehicle, 500 mM VPA, 100 mM MNX or derivatives of VPA (500 mM PBA, 500 mM EBA) were topically applied to shaved back skin of C3H mice daily for 28 d. (A) Gross images of hair re-growth (first row panel), immunohistochemistry of the drug-treated skin with antibody against β-catenin (second row panel), and H&E staining of the drug-treated skin (third row panel). (B) Quantitative histomorphometric analyses were performed for 5 mice for each group. Asterisks denote significant differences between control and test group as measured by t-test with one asterisks being p<0.05, two asterisks being p<0.005, and three asterisks being p<0.0001. Original magnification: A, ×100 (H&E staining) and ×635 (immunohistochemistry).

**Figure 5:**
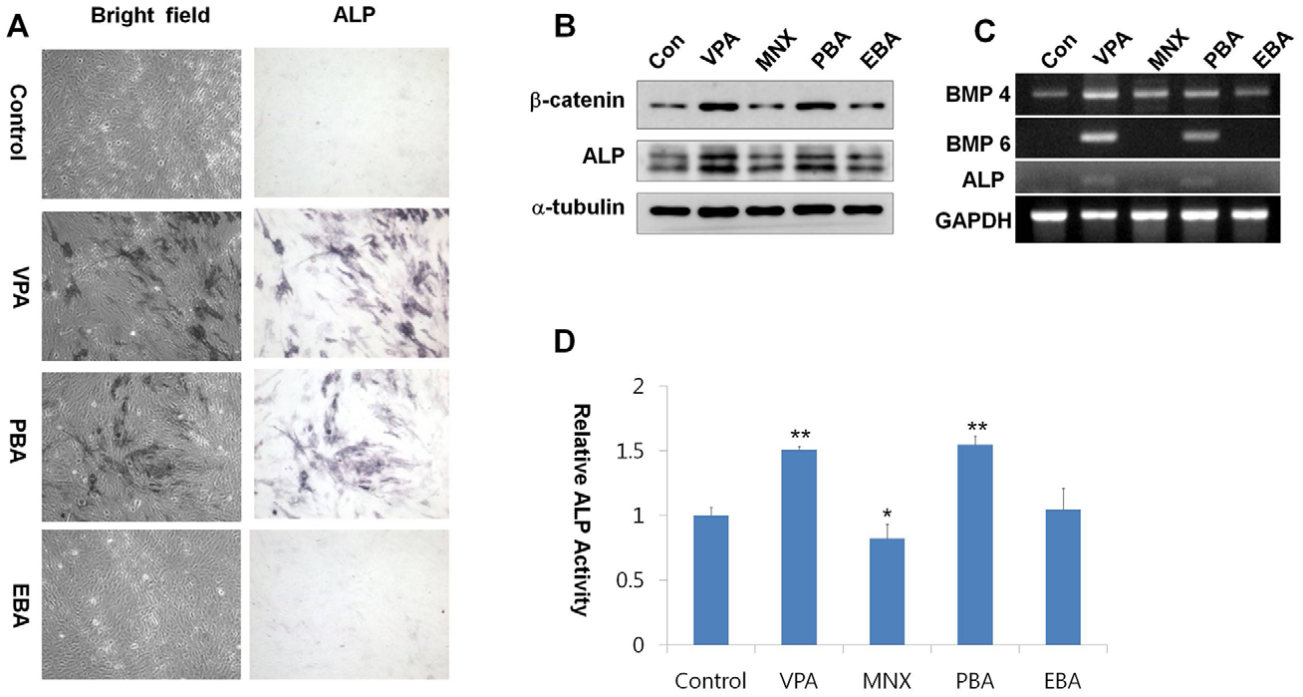
Effects of Wnt/β-catenin pathway activators on the regulation of BMP and alkaline phosphatase activity. Human dermal papilla cells at passage 11 with minimal ALP activity were used to test the ability to recover ALP activity. (A) Morphology (upper panels) and ALP staining patterns (lower panels) of human dermal papilla cells treated with 1 mM VPA, 1 mM PBA, 1mM EBA. Cells were observed under bright-field. (B) Western blotting for β-catenin, ALP, or α-tubulin expression in extracts prepared from drug-treated human dermal papilla cells. (C) RT-PCR analysis of BMP4, BMP6, ALP, and GAPDH expression using total RNA prepared from drug-treated human dermal papilla cells. (D) ALP activity. Asterisks denote significant differences between control and test group as measured by t-test with one asterisk being p<0.05, two asterisks being p<0.005. Original magnification: B, ×100.

**Figure 6:**
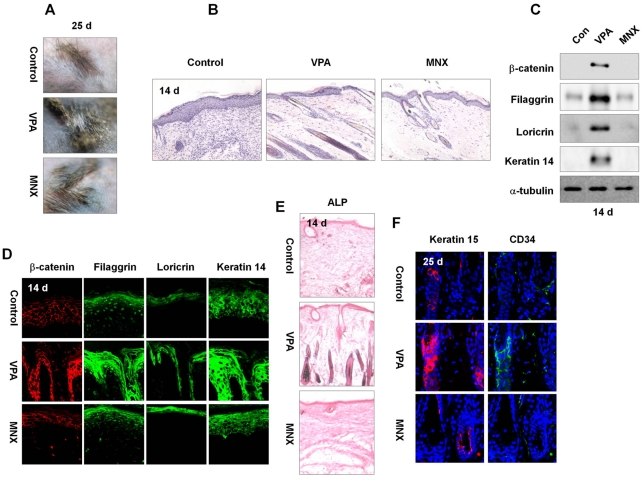
Effects of VPA or MNX on wound-induced hair growth. Four full-thickness excisions (0.2 cm^2^ circular wounds) of skin were made on the backs of 8-wk-old C3H or TOP-Gal transgenic mice, and 500 mM VPA or 100 mM MNX was topically applied daily to the wounds. (A) Gross images of representative wounded back skin of C3H mice 25 d after drug application. (B) H&E staining of wounded skin treated with VPA or MNX. (C, D) Western blot (C) and Immunohistochemical (D) analyses of β-catenin, filaggrin, loricrin and keratin 14 in the wounds. (E) Wounded skins were treated with vehicle, VPA, or MNX for 14 d, and tissue was subjected to ALP staining assays. (F) Immunohistochemical analysis was performed with anti-keratin 15, or -CD34 antibody. Original magnification: B, ×200; C, ×635; D, ×200; E, ×200; F, ×635.

### Immunohistochemistry

Skin tissues were fixed overnight in 4% paraformaldehyde. Paraffin sections (4-µM thick) were deparaffinized and rehydrated. For antigen retrieval, the slides were autoclaved in 10 mM sodium citrate buffer. Sections were pre-incubated in PBS and then blocked in PBS containing 5% bovine serum albumin and 1% goat serum at room temperature for 30 min. The sections were incubated overnight at 4°C with the following dilutions of primary antibodies: anti-β-catenin (BD transduction laboratory, 1∶100), p-Erk (Cell Signaling, 1∶30), p-Akt (Santa Cruz, 1∶50), PCNA (Santa Cruz, 1∶500), filaggrin (Covance, 1∶500), loricrin (Covance, 1∶500), keratin 14 (Covance, 1∶500), BMP4 (R&D systems, 1∶50), Keratin 15 (Thermo Scientific, CA, 1∶200), CD34 (Abcam, 1∶30), nestin (BD Bioscience, 1∶200), Ki67 (Abcam, 1∶500), and keratin 17 (Abcam, 1∶100). The sections were rinsed with PBS and incubated with Alexa Fluor 488- or Alexa Fluor 555-conjugated IgG secondary antibody (Molecular Probes, 1∶400) at room temperature for 1 h and counterstained with DAPI (4′,6-diamidino-2-phenylindole, Boehringer Mannheim, 1∶5000). The fluorescent signals were visualized on a LSM510 META confocal microscope (Carl Zeiss).

### Alkaline Phosphatase Staining

Human dermal papilla cells were plated in 12-well plates at 7.5×10^4^ cells per well. After growth for 24 h, cells were treated with drugs for 72 h, and then fixed in 4% paraformaldehyde for 10 min and washed with PBS. For optimal staining, cells were rinsed in TN buffer (0.1 M Tris-HCl, 0.1 M NaCl, pH 9.5). Cells were incubated in 120 µg/ml 4-nitroblue tetrazolium and 60 µg/ml BCIP (5-bromo-4-chloro-3-indolylphophate) in TN buffer for 30 min. The reaction was stopped by washing with PBS, and the cells were examined under a bright field microscope. Dark blue staining indicates positive signal for ALP. For ALP staining of tissues, 10-µM cryosections were incubated with BCIP and analyzed as above. ALP activity was measured at 405 nm by FLUOstar OPTIMA luminometer.

### X-gal Staining of Tissues

Fresh skin tissue was fixed in 4% paraformaldehyde overnight and then embedded in Tissue Tek O.C.T. compound (Sakura Finetechnical). Cryosections (10-µM) were prepared on coated slides and used for X-gal (5-Bromo-4-chloro-3-indolyl-β-D-galactopyranoside, Gold BioTechnology) staining. The sections were fixed with 0.2% glutaraldehyde for 5 min, washed with PBS for 5 min, and incubated in 1 mg/ml X-gal solution for 24 h at room temperature in a humid environment. The slides were then washed for 5 min with PBS, rinsed with distilled water, and counterstained with Nuclear Fast Red for 5 min.

## Results

### Valproic Acid Promotes Hair Re-Growth and Induces Terminally Differentiated Hair Markers *in Vivo*

We tested the hair re-growth activity of LiCl and VPA, two chemical activators of the Wnt/β-catenin pathway [25], [26]. VPA or LiCl was topically applied daily onto the backs of C3H mice at different concentrations to determine the optimal concentration for each agent. MNX was separately applied as a positive control. The mice treated with 1 M LiCl or 500 mM VPA showed hair growth phenotypes (Figures S1A and S1B). Especially, VPA promoted hair re-growth as efficiently as MNX after 28 d (Figure 1A). The hair follicles of mice treated with VPA or MNX entered anagen phase, whereas hair follicles in the control group treated with vehicle solution remained in telogen phase (Figure 1A, data for different drug treatment times are shown in Figure S2A). The histomorphometrical analyses showed that VPA promoted telogen-anagen transition (Figure 1B). Especially, the hair follicles of mice treated with VPA were transformed to middle- or late-anagen (Figure 1B). Immunohistochemical analysis confirmed that expression of filaggrin and loricrin was increased by VPA or MNX (Figure 2A, data for different drug treatment times are shown in Figures S2B and S2C). We did not observe any significant abnormal phenotypes in the epidermis, hair follicles, or other skin structures aside from hair re-growth following application of VPA or MNX (Figure 1A). In contrast to the epidermis of mouse skin treated with VPA, skin that was treated with LiCl revealed critical abnormal changes including an increase in the thickness of the epidermis (Figure S3), where expression of filaggrin, loricrin, and keratin 14 was also abnormally elevated as shown by immunohistochemistry (Figure S3).

### Valproic Acid Activates the Wnt/β-Catenin Pathway in Addition to the Erk and Akt Pathways

The expression of β-catenin in mouse skin was significantly increased by application of VPA, but only slightly increased by MNX (Figure 2A). MNX is known to promote hair re-growth via the Erk and Akt pathways, which are involved in the regulation of proliferation in dermal papilla cells of the hair follicle [27]. Interestingly, the activities of both Erk and Akt were similarly enhanced by treatment with either VPA or MNX (Figure 2A). The expression level of the proliferation marker PCNA was increased by application of VPA or MNX compared to control skin (Figure 2A). Thus, VPA up-regulates the Wnt/β-catenin pathway in addition to the Erk and Akt pathways, but via a different mechanism.

To examine the short-term effects of VPA on hair re-growth, we analyzed the skin of C3H mice after application of VPA or MNX for 7 d. The thickness of the epidermis increased slightly and the number of hair follicles increased 7 d after application of VPA or MNX (Figure S4A, upper panel). Immunohistochemical analysis showed that keratin14 expression was increased following a 7 d application of VPA or MNX (Figure S4A, lower panel), although the level of keratin14 was not changed 28 d application of VPA or MNX (Figure 2A). Interestingly, VPA, but not MNX, greatly increased the expression of β-catenin in the hair follicles of C3H mice (Figure 2B). We also observed significant induction of ALP in the dermal papilla following application of VPA, but not MNX (Figures 2B and S4B). Moreover, we confirmed specific activation of the Wnt/β-catenin pathway in the pre-cortex regions [4] of the skin of TOP-Gal Wnt reporter mice treated for 7 d with VPA, but not MNX (Figure S4C). Interestingly, Keratin 15 and CD34, the hair follicular stem cell markers, were induced in bulge cells by application of VPA for 7 d (Figure 2C), but not by application of MNX.

### Valproic Acid, but not MNX, Up-Regulates the Wnt/β-Catenin Pathway and ALP Activity in Human Dermal Papilla Cells

To identify whether VPA can activate the Wnt/β-catenin pathway in human systems, we used an *in vitro* culture system of human dermal papilla cells. The expression level of β-catenin was greatly increased by treatment with VPA, but not MNX for 72 h (Figures 3A and S5). Similarly, expression of both BMP4 and ALP was increased by VPA, but not MNX (Figures 3A and S5). We also confirmed significant activation of β-catenin and BMP4 in human dermal papilla cells treated with VPA by immunocytochemistry, and again those changes were not observed following treatment with MNX (Figure 3B). To evaluate the effect of VPA or MNX on the regulation of ALP activity, we used human dermal papilla cells at passage 11 that showed very weak ALP activity. We observed a significant increase in ALP activity following treatment with VPA, but not MNX (Figure 3C). Moreover, the induction of ALP activity by VPA was blocked by noggin, a BMP4 antagonist (Figure 3D). To confirm the role of the Wnt/β-catenin pathway in the activation of ALP, we measured the effects of Wnt3a, BMP4, or epidermal growth factor (EGF) ligand on ALP. Expression of both ALP and β-catenin was significantly increased by treatment with Wnt3a or BMP4 in a concentration-dependent manner, whereas these changes were not significantly induced by treatment with EGF (Figure 3E). The specific activation of ALP by Wnt3a and BMP was also confirmed by a direct enzyme assay (Figure 3F).

### Activators of the Wnt/β-Catenin Pathway Promote Hair Re-Growth *in Vivo*

To confirm the role of the Wnt/β-catenin pathway in hair re-growth, we tested the effects of drugs that regulate the Wnt/β-catenin pathway on hair re-growth in mice. Beryllium chloride (BeCl_2_), LiCl (an alternative GSK3β inhibitor), and several derivatives of VPA including 4-phenyl butyric acid (PBA) and 2-ethyl butyric acid (EBA) were tested for their effects on hair re-growth. PBA or EBA induced hair re-growth after topical application to the back of C3H mice for 28 d (Figure 4A, first row panel). The levels of β-catenin were increased by treatment with PBA, but not EBA (Figure 4A, second row panel). The hair follicles of skin tissues treated with PBA or EBA entered anagen phase as shown by H&E staining (Figure 4A, third row panel). Histomorphometrical analysis revealed that PBA and EBA also induced telogen-anagen transition (Figure 4B). LiCl or BeCl_2_ also induced hair re-growth after 35 d although its hair growing activity was mild (Figure S6A, first row panel). Treatment with LiCl or BeCl_2_ increased the levels of β-catenin and accelerated hair cycle into the anagen phase (Figure S6A, second row panel and third row panel). However, the thickness of the epidermis was increased in skin treated with BeCl_2_ or PBA compared to control skin, as previously described for LiCl application. The expression of filaggrin and loricrin was abnormally increased by application of BeCl_2_, similar to the effect of LiCl (Figure S6B). However, the activities of Erk and Akt were increased by treatment with all of the drugs, including EBA (Figures S6C and S7).

ALP activity was increased by treatment with VPA or PBA, which also up-regulated the Wnt/β-catenin pathway in human dermal papilla (Figure 5A). Interestingly, treatment with EBA, which did not affect the Wnt/β-catenin pathway, did not significantly increase ALP activity (Figure 5A). In addition, the protein and mRNA levels of ALP were increased following treatment with VPA or PBA, which also increased the protein level of β-catenin, but not by treatment with MNX or EBA (Figures 5B, 5C, and S8). Expression of BMP4 mRNA was increased by treatment with VPA, but was only marginally increased by treatment with MNX or PBA and was not induced at all by EBA. Expression of BMP6, which induces the most pronounced effects on ALP activation among the BMPs [28], was markedly increased by either VPA or PBA but not by MNX or EBA (Figure 5C). The specific activation of ALP by VPA or PBA was also confirmed by a direct enzyme assay (Figure 5D). The mouse model showed similar phenotypes to those observed in human dermal papilla cells; the levels of β-catenin in the epidermis and ALP in dermal papillae were significantly increased by application of VPA or PBA for 7 d, but were not changed by application of MNX or EBA (Figures S9A and S9B).

### Valproic Acid Promotes Hair Growth in Cutaneous Wounds in Mice

Activation of the Wnt/β-catenin pathway in epidermal keratinocytes can potentially induce hair growth in mouse skin that is damaged by wounding [29]. To test the effectiveness of VPA on wound-induced hair growth, we daily applied VPA to the wound area (diameter = 0.5 mm) of C3H mice. The presence of epithelial stem cells in hair follicles around wound areas induces spontaneous hair cycling as previously reported [30], and VPA further significantly enhanced hair growth (Figures 6A and S10A) and the transition from telogen phase to anagen phase at the wound site as revealed by histological analysis (Figure 6B). The expression levels of fillaggrin, loricrin, and keratin 14 in wounds was also specifically increased by application of VPA for 14 d by both immunoblot and immunohistochemical analyses (Figures 6C and 6D). Moreover, VPA specifically activated the Wnt/β-catenin pathway during hair growth at wound sites, as shown by increased β-catenin expression (Figures 6C and 6D) and induction of β-galactosidase in newly formed hair follicles of TOP-Gal Wnt reporter mice (Figure S10B; representative mice hairg growth phenotypes by drug application are shown in Figure S10C). Importantly, we also observed an increase in ALP activity in the hair follicles following application of VPA (Figure 6E). Keratin 15 and CD34, the hair follicular stem cell markers, were increased after 25 d of VPA application to the wounds (Figure 6F).

## Discussion

VPA is an antiepileptic drug frequently prescribed due to its safety and effectiveness [10], [11]. Prolonged use of VPA resulted in several side effects including hair loss by oral intake; these adverse effects are attributed to zinc and biotinidase depletion [31]. We did not observe hair re-growth effects when VPA was orally administered to C57BL/6 mice (Figures S11A and S11B). However, topical application of VPA significantly promoted hair formation in murine models. The levels of β-catenin in the mice skin were specifically increased by topical application of VPA (Figure S11C).

In this study, we demonstrated that GSK3β inhibitors that activate the Wnt/β-catenin pathway [17], [18], [25], [32] could potentially be developed as drugs to treat hair loss and baldness involving defects in hair follicles. Among these, VPA was identified as the most potent hair re-growth factor without causing skin abnormalities in mice. Alternative inhibitors of GSK3β, LiCl or BeCl_2_, also stimulated hair re-growth and returned the hair cycle to the anagen phase, but abnormally increased the thickness of the epidermis with hyper-activation of terminally differentiated epidermal markers. In contrast to the epidermis of mouse skin treated with other GSK3β inhibitor, skin of C3H mice treated with VPA didn't reveal any significant abnormal phenotypes in the epidermis. We found that ALP is a highly credible marker for activation of the Wnt/β-catenin pathway, and importance of the Wnt/β-catenin pathway in the activation of ALP was confirmed by the demonstration that ALP was not regulated by MNX or EBA, which did not induce expression of β-catenin and BMP4. It is known that VPA stimulates neuronal differentiation of neural progenitors through the induction of BMP4 [33], [34], and the effect of BMP4 on hair-inducing activity was also previously reported [28]. Our study reporting that BMP4 plays a role as an activator of ALP further confirms the importance of the Wnt/β-catenin pathway in hair re-growth.

Although the relative effect was small compared to VPA or PBA, EBA (which did not activate β-catenin and BMP4 or ALP), still induced hair formation. These results indicate that the hair-inducing activity of EBA may be independent of the Wnt/β-catenin pathway, and in fact we confirmed that EBA induced activation of Erk and Akt, which are in turn involved in keratinocyte proliferation. Interestingly, VPA induced expression of the hair follicular stem cell markers ketatin 15 and CD34 during hair formation and wound-induced growth. VPA is known to induce CD34 expression and enhance stemness [35], [36]. The bald scalps of men with androgenetic alopecia lack CD200-rich, CD34-positive hair follicle progenitor cells, and have a defect in conversion of hair follicle stem cells to progenitor cells, which play a role in the pathogenesis of androgenetic alopecia [37]. The results of our study indicate that small molecules that activate the Wnt/β-catenin pathway, such as VPA, can potentially be applied for the development of drugs to accelerate hair cycle and stimulate hair re-growth.

## Supporting Information

Figure S1 **Hair re-growth following topical application of LiCl, VPA, or MNX to the mice skin.** Back skin of 8-wk-old male C3H mice were shaved, vehicle or various concentrations of LiCl, VPA, or MNX were applied topically for 35 d. (A) Gross images of mice treated with LiCl, VPA, or MNX. (B) Enlarged images of representative skin from Figure S1A.
(TIF)

Figure S2 **Effects of VPA or MNX on β-catenin, epidermal differentiation, and proliferation markers in mouse skin.** C3H mice were treated with VPA or MNX for 7, 14, or 21 d. H&E staining and immunohistochemical analyses were performed as described in Figure 1 and 2. Original magnification: H&E, ×200; immunohistochemistry, ×635.
(TIF)

Figure S3 **Effects of LiCl on epidermal differentiation markers, β-catenin, PCNA, and activities of Erk and Akt in mouse skin.** LiCl-treated skin tissue was excised and subjected to H&E staining, ALP staining, or immunohistochemical analysis to detect filaggrin, loricrin, keratin14, β-catenin, p-Erk, p-Akt, and PCNA as described in Figures 1 and 2. Original magnification: H&E, ×100; immunohistochemistry, ×635; ALP staining, ×200.
(TIF)

Figure S4 **Effects of VPA or MNX on expression of keratin14, ALP or β-galactosidase activities monitoring the Wnt/β-catenin activities in mice skin.** C3H mice were treated with VPA or MNX for 7 d. (A) H&E staining (upper panel) and immunohistochemical analysis for keratin 14 (lower panel) in treated skin. (B) ALP staining of control, VPA- or MNX-treated mice skin. (C) TOP-GAL transgenic mice tissues treated with vehicle, VPA, or MNX for 7 d were subjected to the X-gal staining to examine regulation of the Wnt/β-catenin pathway. Blue region of hair follicles represent positive Wnt/β-catenin signaling. Original magnification: A, ×100; B, ×200; C, ×400 (left panel) and ×200 (right panel).
(TIF)

Figure S5 **Effects of VPA and MNX on the activation status of the Wnt/β-catenin pathway and ALP in human dermal papilla cells.** Human dermal papilla cells were grown in DMEM supplemented with 10% heat-inactivated FBS and were treated with 1 mM VPA or 100 µM MNX for 72 h. Western blotting was performed in VPA or MNX-treated human dermal papilla cells. The relative expression of each protein such as β-catenin, BMP4, and ALP was calculated as the ratio of each protein expression level to α-tubulin level. The software used for the quantification was Multi-Gauge V 3.0 (Fujifilm). Asterisks denote significant differences between control and test groups as measured by t-test with one asterik being p<0.05, two asteriks being p<0.005, and three asteriks being p<0.0001.
(TIF)

Figure S6 **Effects of Wnt/β-catenin pathway activators on hair re-growth in mouse skin.** The back skin of C3H mice was shaved and treated with topical application of vehicle, alternative GSK3β inhibitor (1 M LiCl, 200 mM BeCl_2_) daily for 35 d. (A) Gross images of hair re-growth (first row panel), immunohistochemistry of LiCl or BeCl_2_-treated skin with antibody against β-catenin (second row panel), and H&E staining of LiCl or BeCl_2_-treated skin (third row panel). (B) Immunohistochemical analysis for filaggrin, loricrin, and keratin14 in LiCl or BeCl_2_-treated skin. (C) Immunohistochemical analysis for p-Erk and p-Akt in LiCl or BeCl_2_-treated skin. Original magnification: A, ×100 (H&E staining) and ×400 (immunohistochemistry); B, ×635; C, ×635.
(TIF)

Figure S7 **Effects of VPA, MNX and derivatives of VPA on epidermal differentiation markers on mouse skin.** The back skin of C3H mice was shaved and treated with topical application of the vehicle, 500 mM VPA, 100 Mm MNX, or derivatives of VPA (500 mM PBA, 500 mM EBA) daily for 28 d. Immunohistochemical analysis for filaggrin, loricrin, keratin14, β-catenin, p-Erk, p-Akt, and PCNA in the drug-treated skin. Original magnification: ×635.
(TIF)

Figure S8 **Effects of Wnt/β-catenin pathway activators on the activation status of the Wnt/β-catenin pathway and ALP in human dermal papilla cells.** Human dermal papilla cells were treated with 1 mM VPA, 100 µM MNX, 1 mM PBA, or 1 mM EBA for 72 h. The relative expression of β-catenin or ALP was calculated as the ratio of each protein level to α-tubulin level. The software used for the quantification was Multi-Gauge V 3.0 (Fujifilm). Asterisks denote the significant differences between control and test groups as measured by t-test with one asterisk being p<0.05, two asterisks being p<0.005, and three asterisks being p<0.0001.
(TIF)

Figure S9 **Effects of VPA, MNX, PBA, and EBA on the activation of Wnt/β-catenin pathway, ALP activity** ***in vivo.*** After topical application of 500 mM VPA, 100 mM MNX, 500 mM PBA, or 500 mM EBA onto the backs of C3H mice for 7 d, the skin tissue was excised from the treated area for immunohistochemistry and ALP staining. (A) Immunohistochemical staining for β-catenin or keratin 14. (B) Immunohistochemical analysis for β-catenin (upper panel) and ALP staining (lower panel) of hair follicles of skin treated with vehicle, VPA, MNX, PBA, or EBA. Dark blue regions in the dermal papilla of hair follicles (red arrows) represent positive staining for ALP activity. Original magnification: A, ×635; B, ×635 (immunohistochemistry) and ×400 (ALP staining).
(TIF)

Figure S10 **Effects of VPA and MNX on wound-induced hair growth and activation of the Wnt/β-catenin signaling** ***in vivo*** **.** Four full-thickness skin excisions (0.2 cm^2^ circular wounds) were made on the backs of 8-wk-old C3H mice, and 500 mM VPA or 100 mM MNX was topically applied daily. (A) Gross images of the wounded back skin of C3H mice 25 d after application of drugs. Right panels are magnified representative images. (B) Wounded skins in TOP-GAL transgenic mice were treated with vehicle, VPA, or MNX for 14 d, and tissue was subjected to X-gal staining. (C) Gross images of the wounded back skin of TOP-gal mice 14 d after application of drugs. Original magnification: B, ×200.
(TIF)

Figure S11 **Effects of VPA or MNX by oral administration on hair re-growth.** (A) Gross images of C57BL/6 mice orally administered by VPA or MNX. The back skin of C57BL/6 mice was shaved and VPA or MNX was orally administered at a dose of 200 mg/kg for 35 d. (B) H&E staining of mice skin orally administered or topically applied by VPA. VPA was orally administered at a dose of 200 mg/kg or topically applied at 500 mM for 35 d. (C) Immunohistochemical analysis for β-catenin, filaggirn, loricrin, and keratin14 in mice skin orally or topically applied by VPA. Original magnification: B, ×100; C, ×635.
(TIF)
