# Supplementary figures and images for: Valproic Acid Induces Hair Regeneration in Murine Model and Activates Alkaline Phosphatase Activity in Human Dermal Papilla Cells
Source: PLoS One. 2012 Apr 10;7(4):e34152. doi: 10.1371/journal.pone.0034152 (PMC3323655; doi:10.1371/journal.pone.0034152)

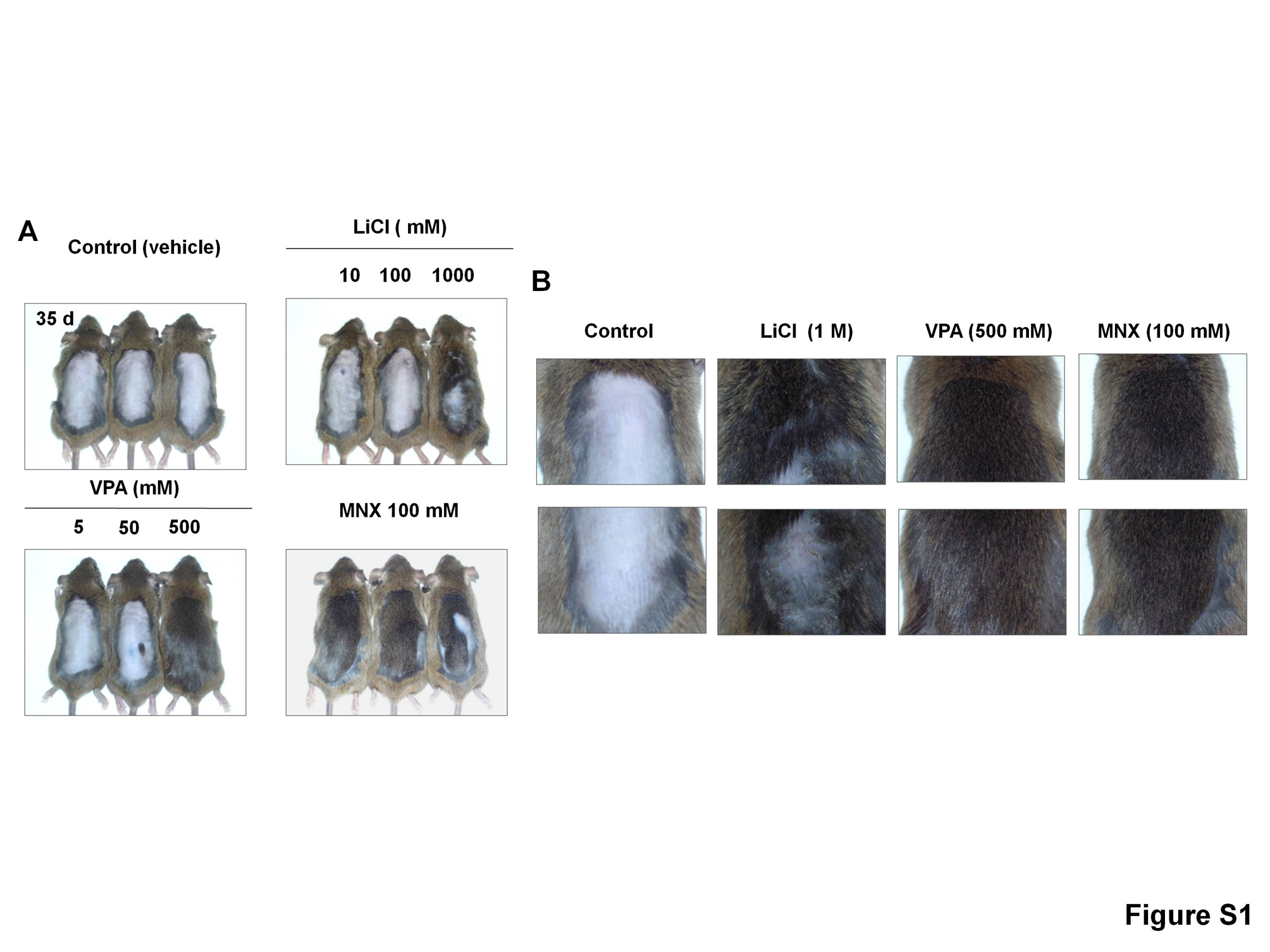

Supplement: Figure S1 — Hair re-growth following topical application of LiCl, VPA, or MNX to the mice skin. Back skin of 8-wk-old male C3H mice were shaved, vehicle or various concentrations of LiCl, VPA, or MNX were applied topically for 35 d. (A) Gross images of mice treated with LiCl, VPA, or MNX. (B) Enlarged images of representative skin from Figure S1A. (TIF) [file pone.0034152.s001.tif]

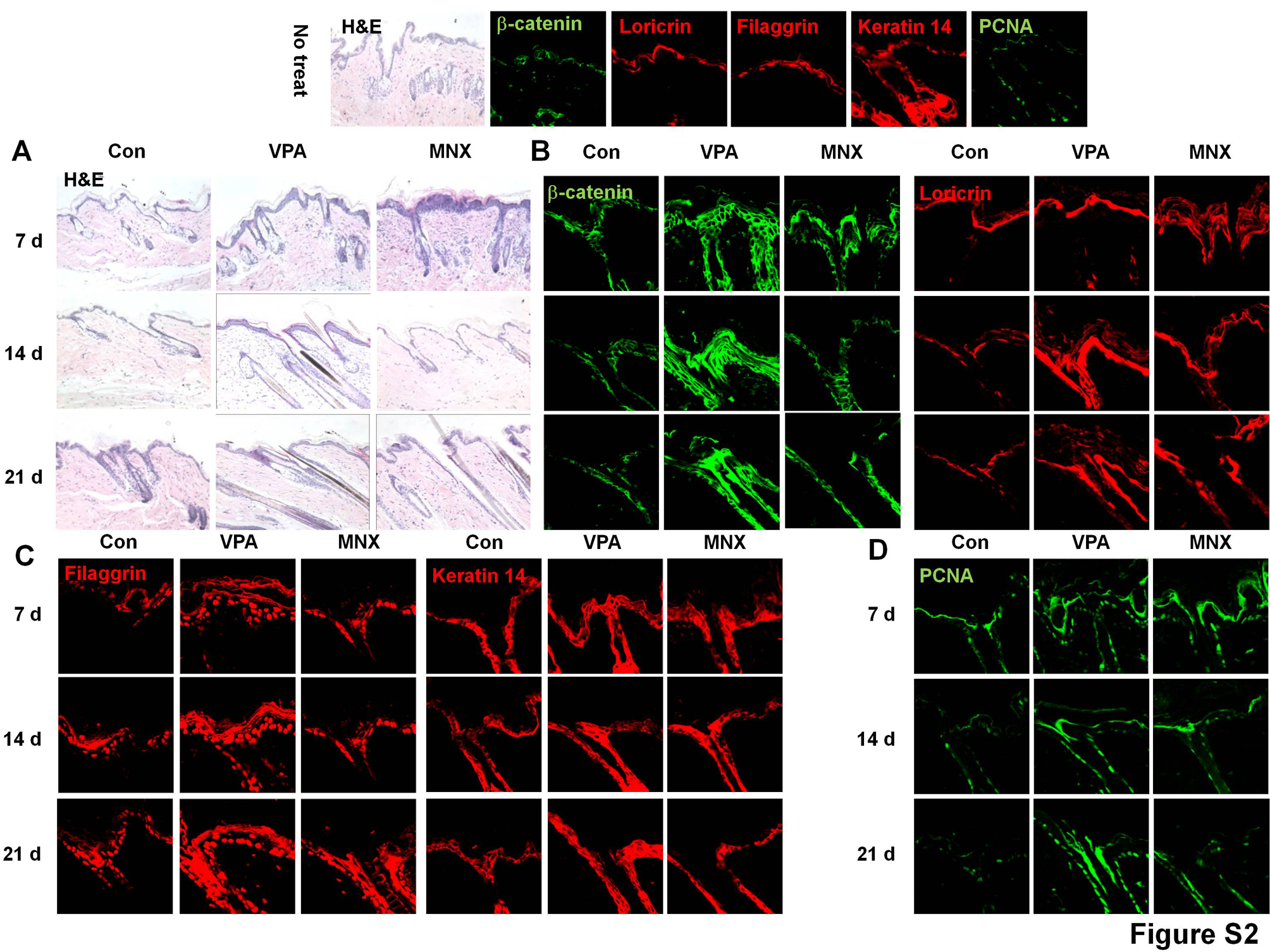

Supplement: Figure S2 — Effects of VPA or MNX on β-catenin, epidermal differentiation, and proliferation markers in mouse skin. C3H mice were treated with VPA or MNX for 7, 14, or 21 d. H&E staining and immunohistochemical analyses were performed as described in Figure 1 and 2. Original magnification: H&E, ×200; immunohistochemistry, ×635. (TIF) [file pone.0034152.s002.tif]

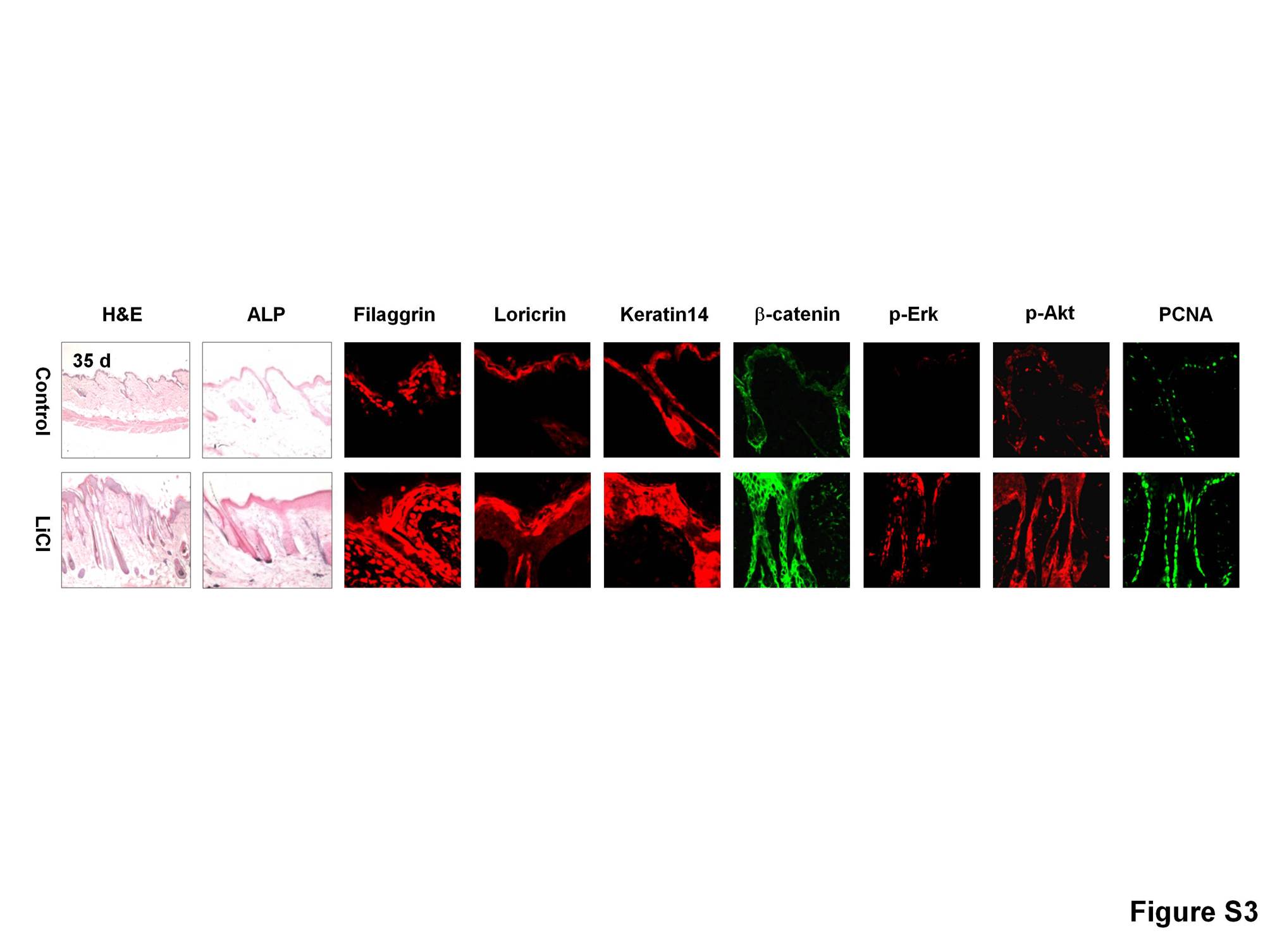

Supplement: Figure S3 — Effects of LiCl on epidermal differentiation markers, β-catenin, PCNA, and activities of Erk and Akt in mouse skin. LiCl-treated skin tissue was excised and subjected to H&E staining, ALP staining, or immunohistochemical analysis to detect filaggrin, loricrin, keratin14, β-catenin, p-Erk, p-Akt, and PCNA as described in Figures 1 and 2. Original magnification: H&E, ×100; immunohistochemistry, ×635; ALP staining, ×200. (TIF) [file pone.0034152.s003.tif]

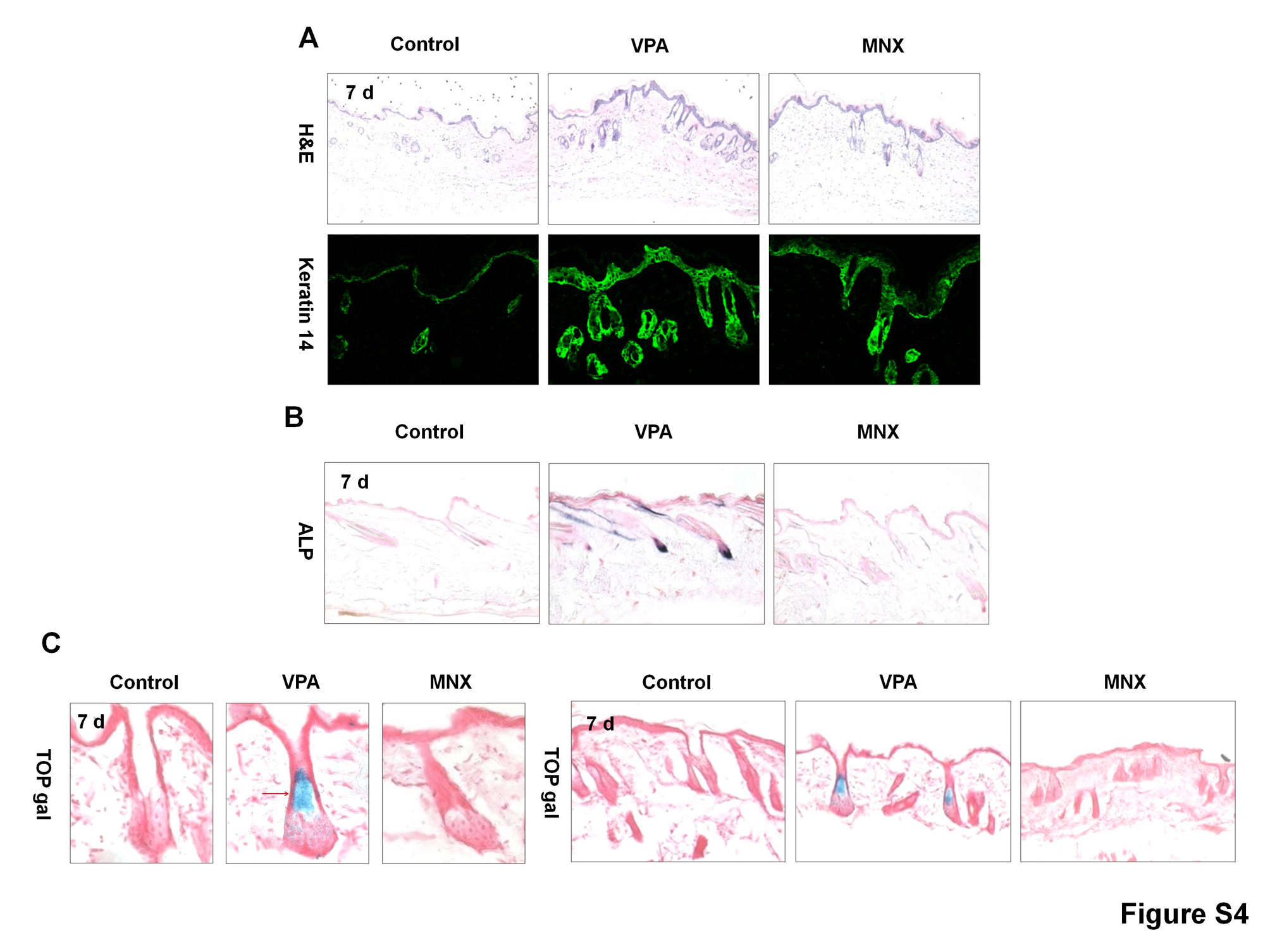

Supplement: Figure S4 — Effects of VPA or MNX on expression of keratin14, ALP or β-galactosidase activities monitoring the Wnt/β-catenin activities in mice skin. C3H mice were treated with VPA or MNX for 7 d. (A) H&E staining (upper panel) and immunohistochemical analysis for keratin 14 (lower panel) in treated skin. (B) ALP staining of control, VPA- or MNX-treated mice skin. (C) TOP-GAL transgenic mice tissues treated with vehicle, VPA, or MNX for 7 d were subjected to the X-gal staining to examine regulation of the Wnt/β-catenin pathway. Blue region of hair follicles represent positive Wnt/β-catenin signaling. Original magnification: A, ×100; B, ×200; C, ×400 (left panel) and ×200 (right panel). (TIF) [file pone.0034152.s004.tif]

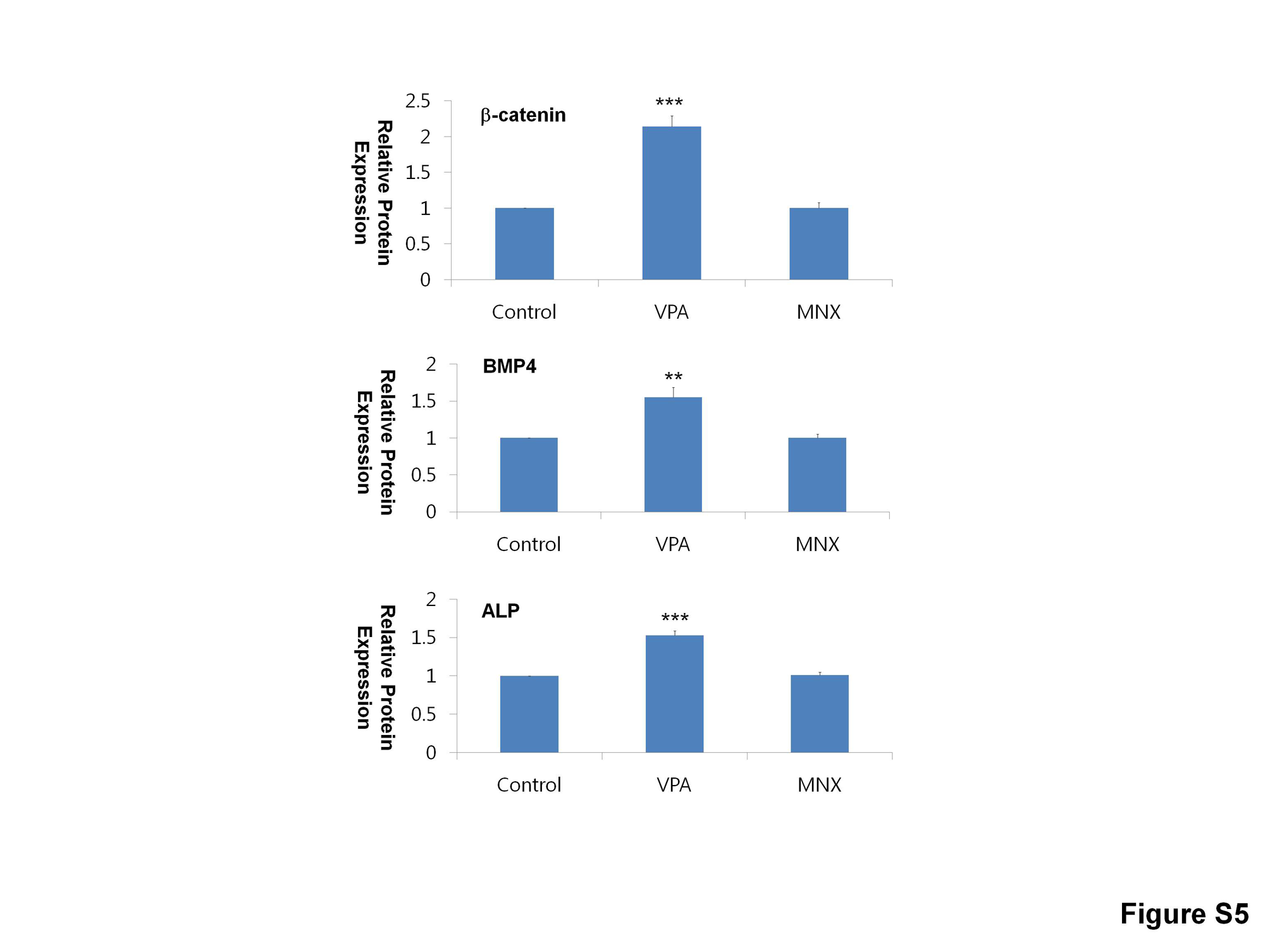

Supplement: Figure S5 — Effects of VPA and MNX on the activation status of the Wnt/β-catenin pathway and ALP in human dermal papilla cells. Human dermal papilla cells were grown in DMEM supplemented with 10% heat-inactivated FBS and were treated with 1 mM VPA or 100 µM MNX for 72 h. Western blotting was performed in VPA or MNX-treated human dermal papilla cells. The relative expression of each protein such as β-catenin, BMP4, and ALP was calculated as the ratio of each protein expression level to α-tubulin level. The software used for the quantification was Multi-Gauge V 3.0 (Fujifilm). Asterisks denote significant differences between control and test groups as measured by t-test with one asterik being p<0.05, two asteriks being p<0.005, and three asteriks being p<0.0001. (TIF) [file pone.0034152.s005.tif]

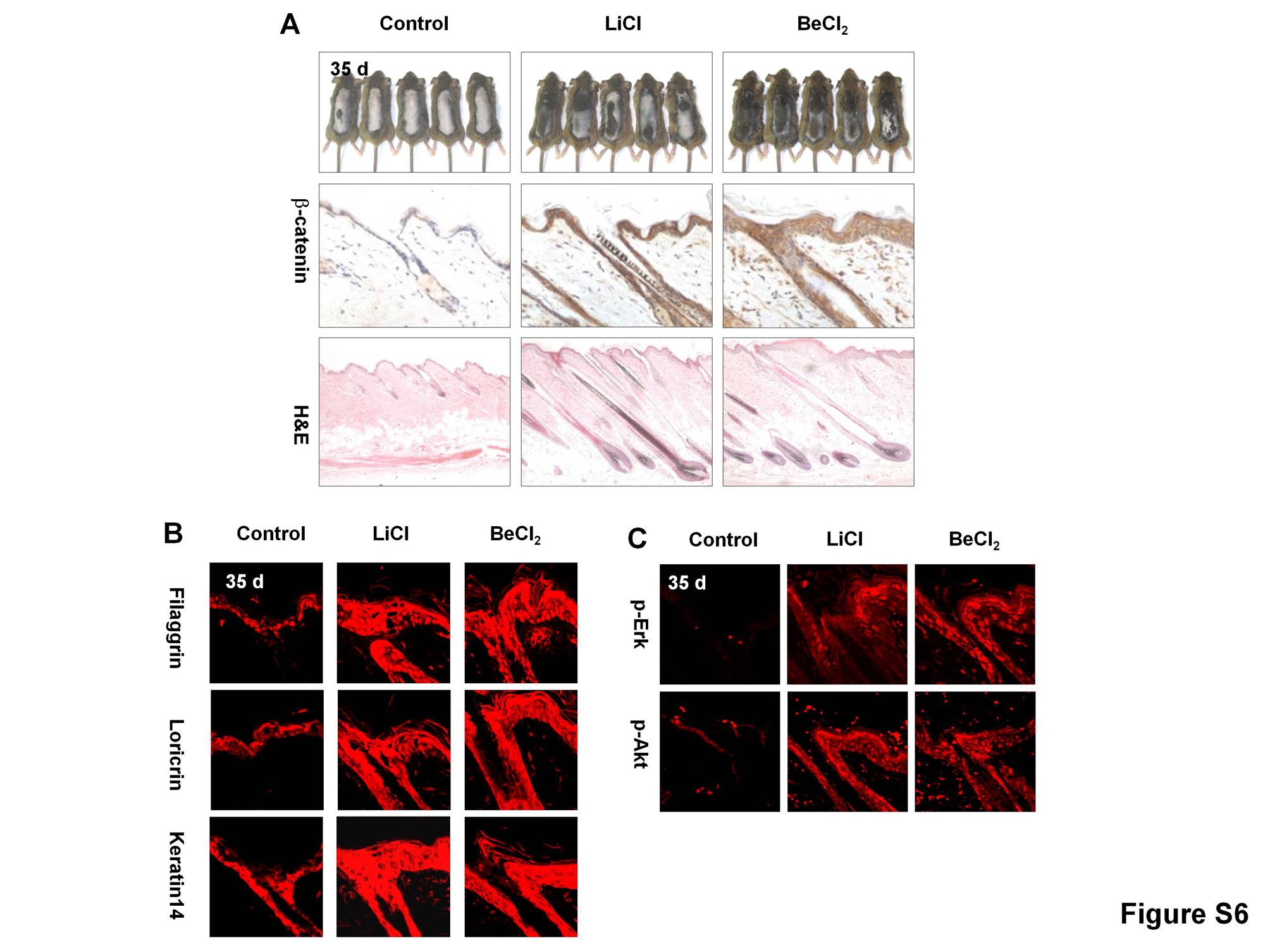

Supplement: Figure S6 — Effects of Wnt/β-catenin pathway activators on hair re-growth in mouse skin. The back skin of C3H mice was shaved and treated with topical application of vehicle, alternative GSK3β inhibitor (1 M LiCl, 200 mM BeCl2) daily for 35 d. (A) Gross images of hair re-growth (first row panel), immunohistochemistry of LiCl or BeCl2-treated skin with antibody against β-catenin (second row panel), and H&E staining of LiCl or BeCl2-treated skin (third row panel). (B) Immunohistochemical analysis for filaggrin, loricrin, and keratin14 in LiCl or BeCl2-treated skin. (C) Immunohistochemical analysis for p-Erk and p-Akt in LiCl or BeCl2-treated skin. Original magnification: A, ×100 (H&E staining) and ×400 (immunohistochemistry); B, ×635; C, ×635. (TIF) [file pone.0034152.s006.tif]

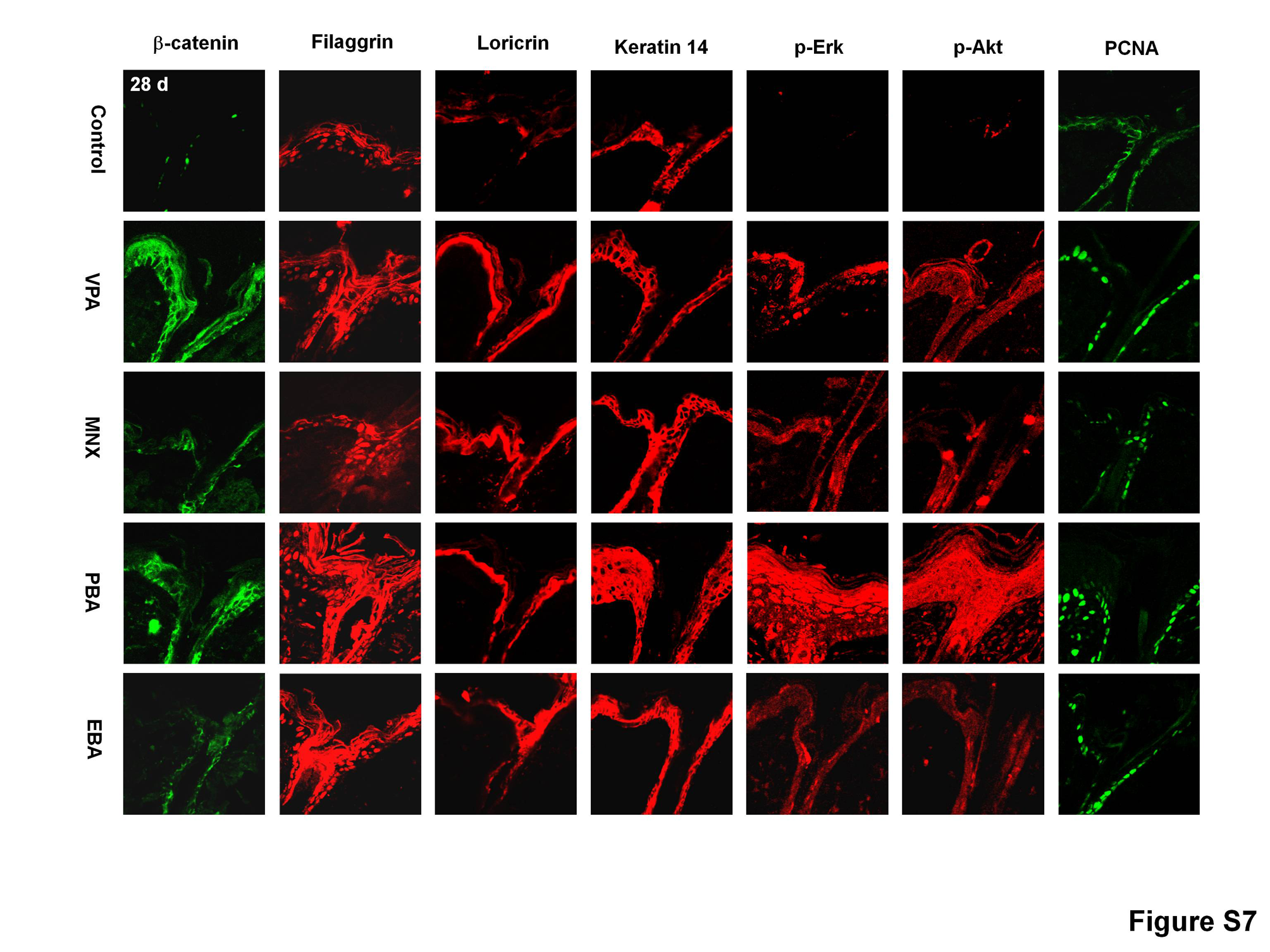

Supplement: Figure S7 — Effects of VPA, MNX and derivatives of VPA on epidermal differentiation markers on mouse skin. The back skin of C3H mice was shaved and treated with topical application of the vehicle, 500 mM VPA, 100 Mm MNX, or derivatives of VPA (500 mM PBA, 500 mM EBA) daily for 28 d. Immunohistochemical analysis for filaggrin, loricrin, keratin14, β-catenin, p-Erk, p-Akt, and PCNA in the drug-treated skin. Original magnification: ×635. (TIF) [file pone.0034152.s007.tif]

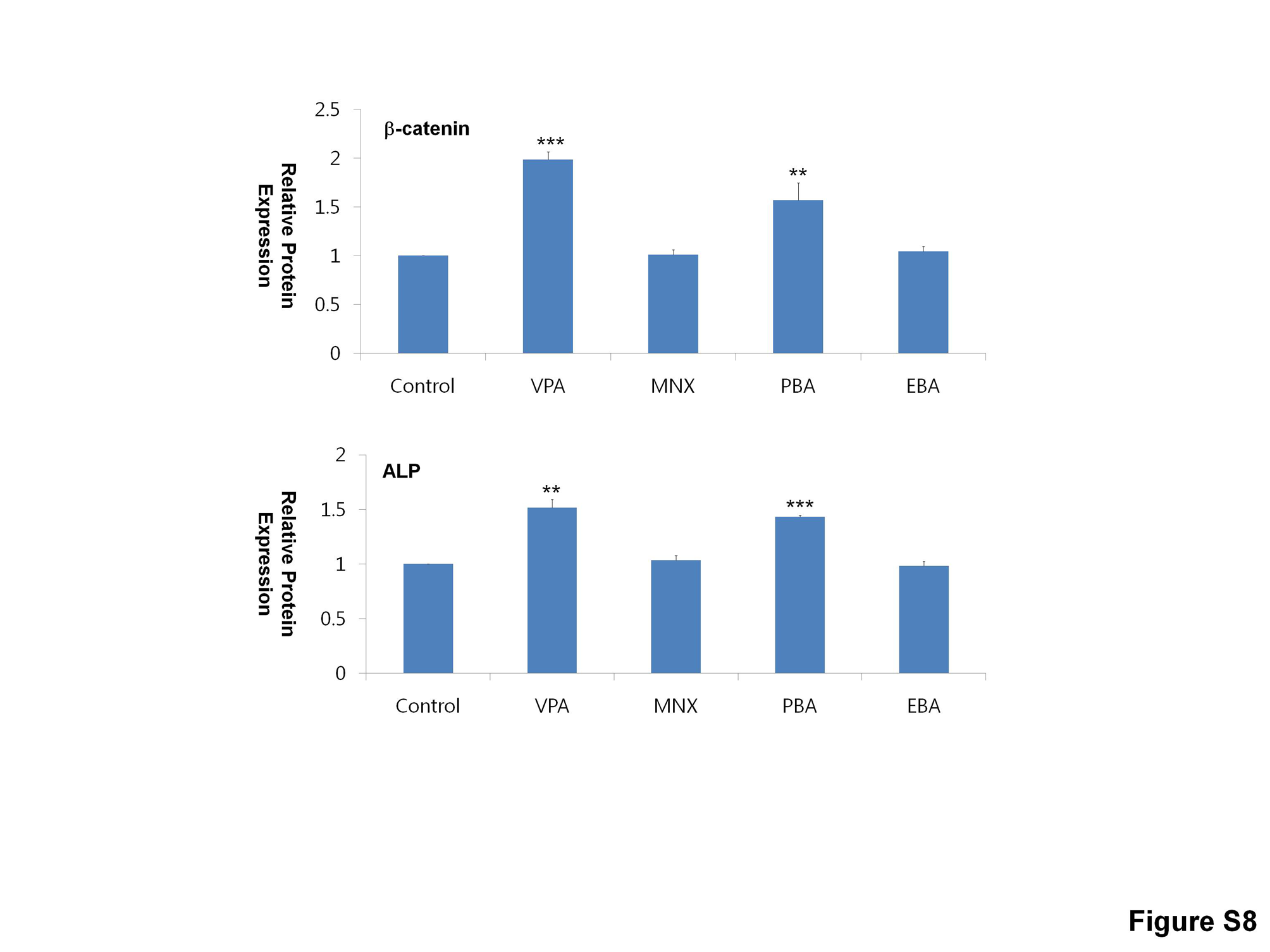

Supplement: Figure S8 — Effects of Wnt/β-catenin pathway activators on the activation status of the Wnt/β-catenin pathway and ALP in human dermal papilla cells. Human dermal papilla cells were treated with 1 mM VPA, 100 µM MNX, 1 mM PBA, or 1 mM EBA for 72 h. The relative expression of β-catenin or ALP was calculated as the ratio of each protein level to α-tubulin level. The software used for the quantification was Multi-Gauge V 3.0 (Fujifilm). Asterisks denote the significant differences between control and test groups as measured by t-test with one asterisk being p<0.05, two asterisks being p<0.005, and three asterisks being p<0.0001. (TIF) [file pone.0034152.s008.tif]

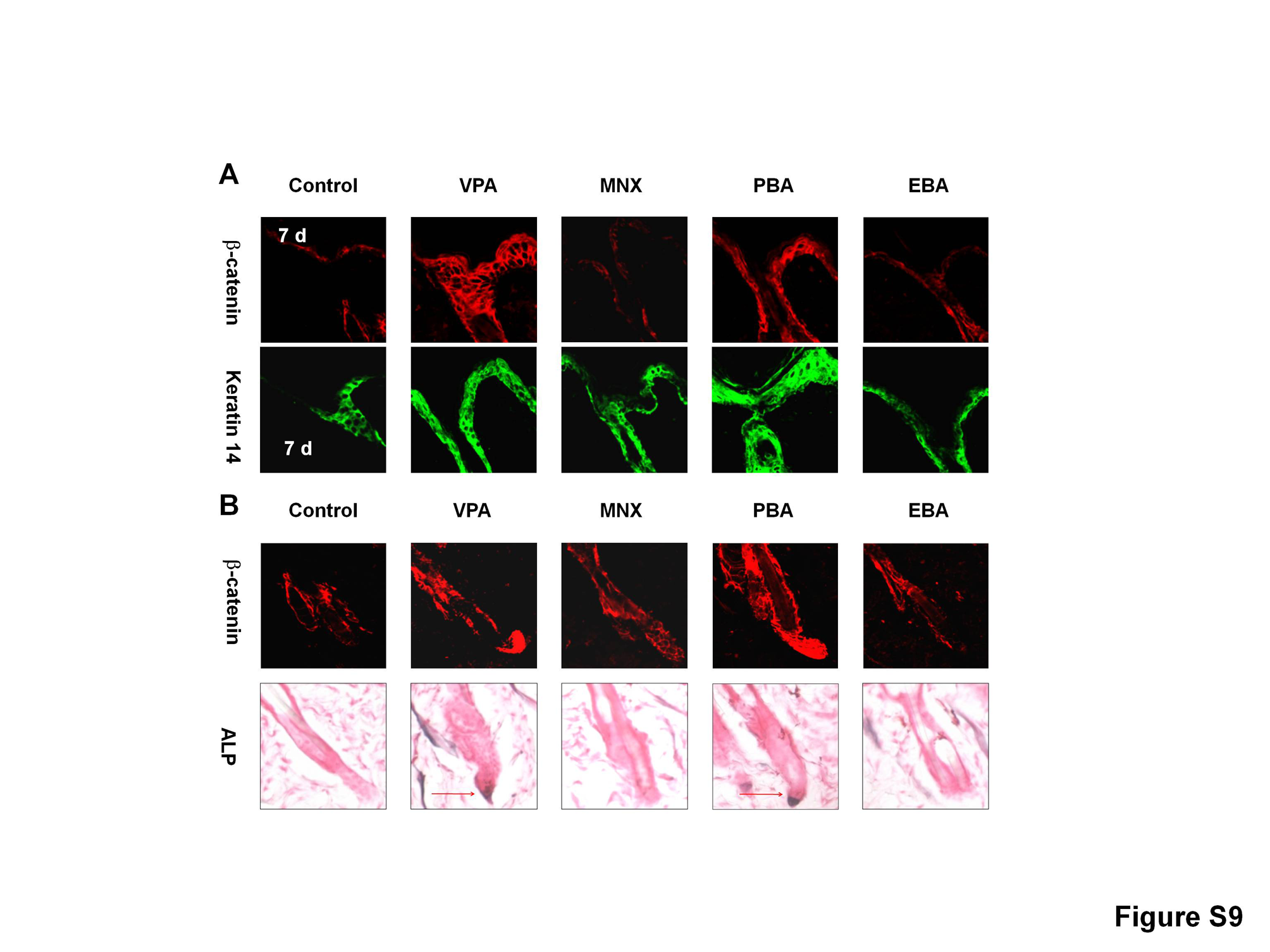

Supplement: Figure S9 — Effects of VPA, MNX, PBA, and EBA on the activation of Wnt/β-catenin pathway, ALP activity in vivo. After topical application of 500 mM VPA, 100 mM MNX, 500 mM PBA, or 500 mM EBA onto the backs of C3H mice for 7 d, the skin tissue was excised from the treated area for immunohistochemistry and ALP staining. (A) Immunohistochemical staining for β-catenin or keratin 14. (B) Immunohistochemical analysis for β-catenin (upper panel) and ALP staining (lower panel) of hair follicles of skin treated with vehicle, VPA, MNX, PBA, or EBA. Dark blue regions in the dermal papilla of hair follicles (red arrows) represent positive staining for ALP activity. Original magnification: A, ×635; B, ×635 (immunohistochemistry) and ×400 (ALP staining). (TIF) [file pone.0034152.s009.tif]

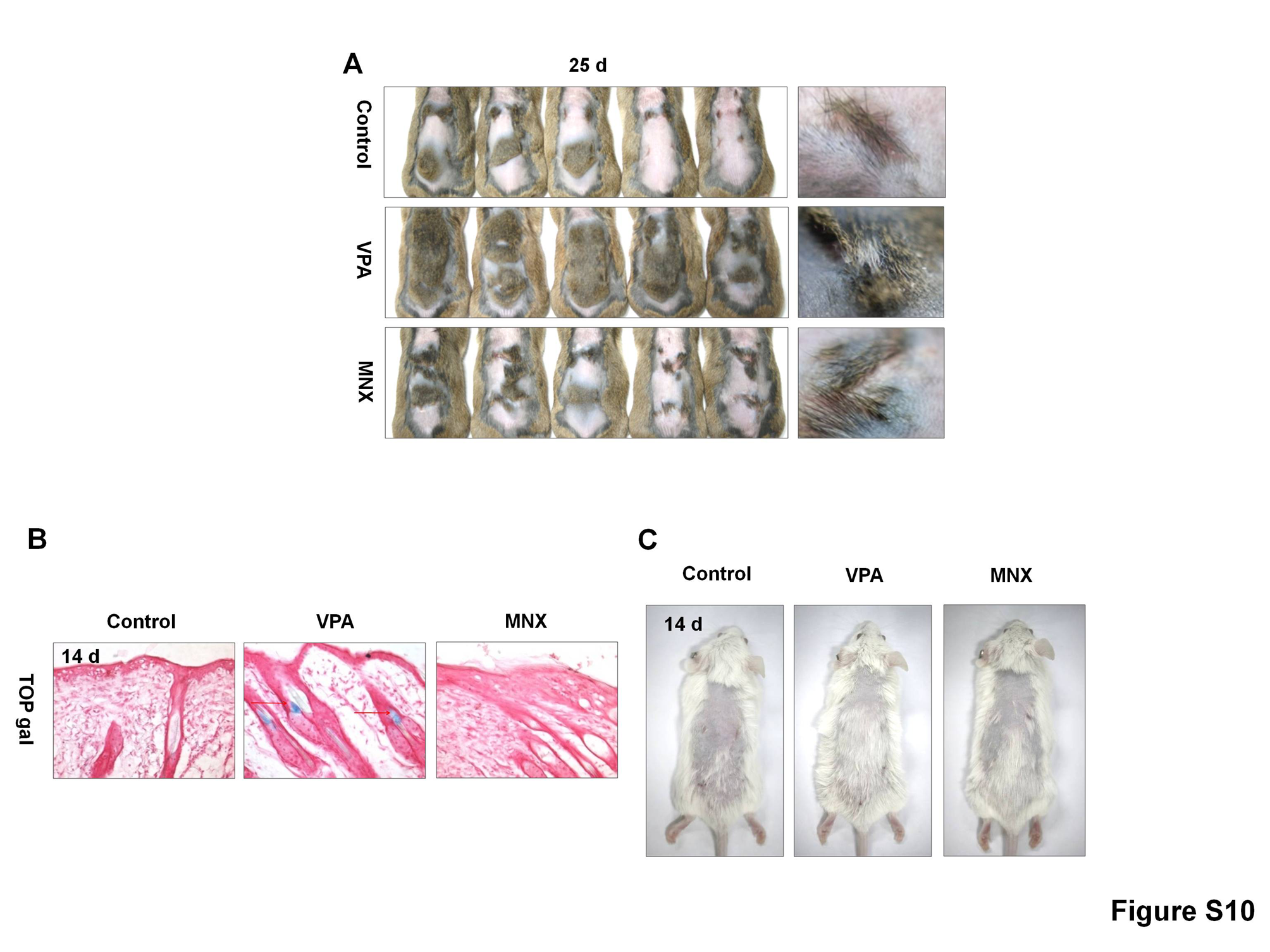

Supplement: Figure S10 — Effects of VPA and MNX on wound-induced hair growth and activation of the Wnt/β-catenin signaling in vivo . Four full-thickness skin excisions (0.2 cm2 circular wounds) were made on the backs of 8-wk-old C3H mice, and 500 mM VPA or 100 mM MNX was topically applied daily. (A) Gross images of the wounded back skin of C3H mice 25 d after application of drugs. Right panels are magnified representative images. (B) Wounded skins in TOP-GAL transgenic mice were treated with vehicle, VPA, or MNX for 14 d, and tissue was subjected to X-gal staining. (C) Gross images of the wounded back skin of TOP-gal mice 14 d after application of drugs. Original magnification: B, ×200. (TIF) [file pone.0034152.s010.tif]

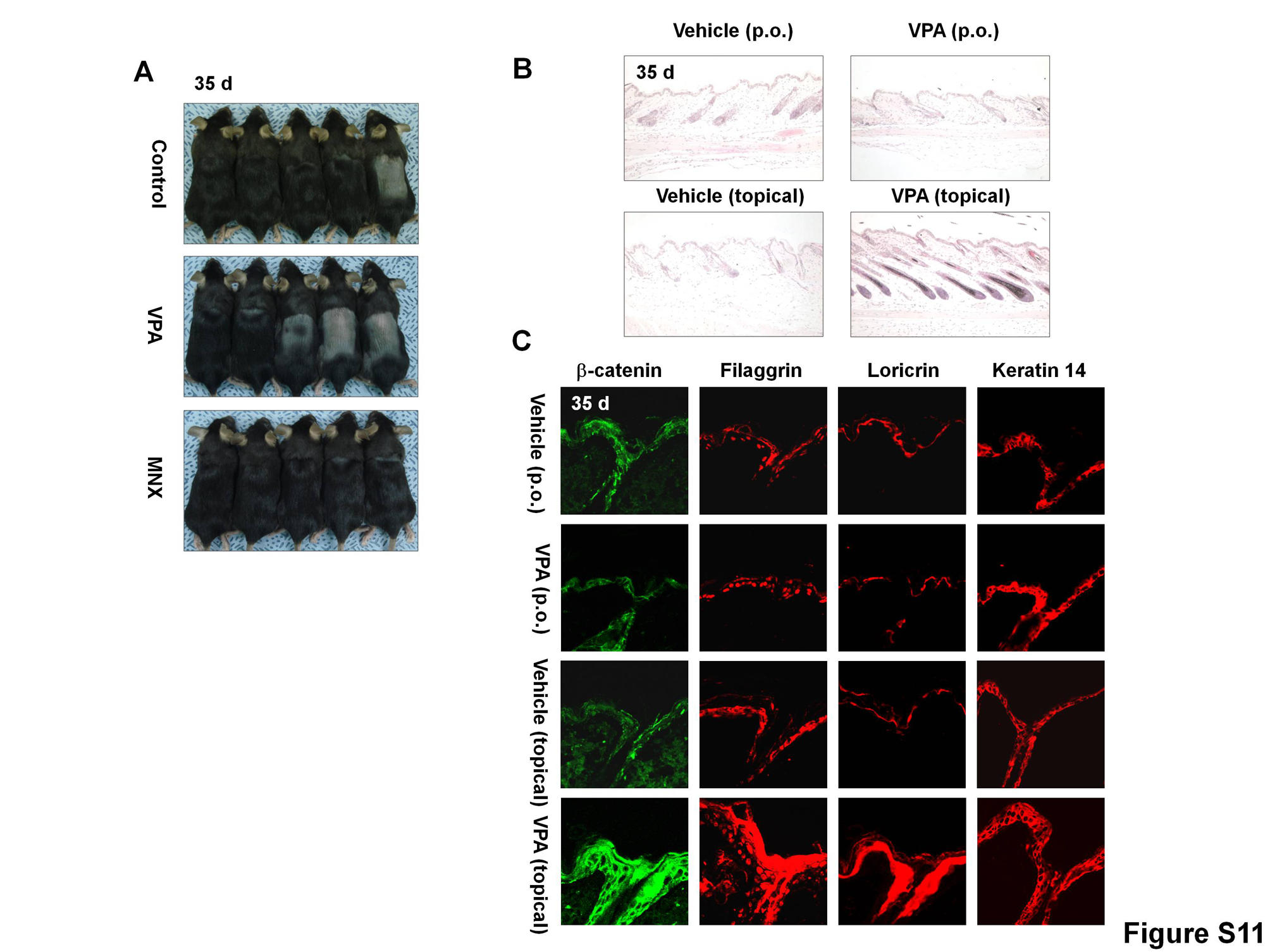

Supplement: Figure S11 — Effects of VPA or MNX by oral administration on hair re-growth. (A) Gross images of C57BL/6 mice orally administered by VPA or MNX. The back skin of C57BL/6 mice was shaved and VPA or MNX was orally administered at a dose of 200 mg/kg for 35 d. (B) H&E staining of mice skin orally administered or topically applied by VPA. VPA was orally administered at a dose of 200 mg/kg or topically applied at 500 mM for 35 d. (C) Immunohistochemical analysis for β-catenin, filaggirn, loricrin, and keratin14 in mice skin orally or topically applied by VPA. Original magnification: B, ×100; C, ×635. (TIF) [file pone.0034152.s011.tif]
